# The Risk of Congenital Heart Anomalies Following Prenatal Exposure to Serotonin Reuptake Inhibitors—Is Pharmacogenetics the Key?

**DOI:** 10.3390/ijms17081333

**Published:** 2016-08-13

**Authors:** Aizati N. A. Daud, Jorieke E. H. Bergman, Wilhelmina S. Kerstjens-Frederikse, Henk Groen, Bob Wilffert

**Affiliations:** 1 Department of Pharmacy, Unit of PharmacoTherapy, -Epidemiology and -Economics, University of Groningen, 9713AV Groningen, The Netherlands; b.wilffert@rug.nl; 2 School of Pharmaceutical Sciences, Discipline of Clinical Pharmacy, Universiti Sains Malaysia, 11800 Penang, Malaysia; 3 Department of Genetics, University Medical Center Groningen, University of Groningen, 9713AV Groningen, The Netherlands; j.e.h.van.kammen@umcg.nl (J.E.H.B.); w.s.kerstjens@umcg.nl (W.S.K.-F.); 4 Department of Epidemiology, University Medical Centre Groningen, University of Groningen, 9713AV Groningen, The Netherlands; h.groen01@umcg.nl; 5 Department of Clinical Pharmacy and Pharmacology, University Medical Center Groningen, University of Groningen, 9713AV Groningen, The Netherlands

**Keywords:** congenital heart defects, heart abnormalities, antidepressive agents, teratogenesis, serotonin reuptake inhibitors, drug-induced birth defects

## Abstract

Serotonin reuptake inhibitors (SRIs) are often prescribed during pregnancy. Previous studies that found an increased risk of congenital anomalies, particularly congenital heart anomalies (CHA), with SRI use during pregnancy have created concern among pregnant women and healthcare professionals about the safety of these drugs. However, subsequent studies have reported conflicting results on the association between CHA and SRI use during pregnancy. These discrepancies in the risk estimates can potentially be explained by genetic differences among exposed individuals. In this review, we explore the potential pharmacogenetic predictors involved in the pharmacokinetics and mechanism of action of SRIs, and their relation to the risk of CHA. In general, the risk is dependent on the maternal concentration of SRIs and the foetal serotonin level/effect, which can be modulated by the alteration in the expression and/or function of the metabolic enzymes, transporter proteins and serotonin receptors involved in the serotonin signalling of the foetal heart development. Pharmacogenetics might be the key to understanding why some children exposed to SRIs develop a congenital heart anomaly and others do not.

## 1. Introduction 

The use of antidepressants during pregnancy, particularly the use of selective serotonin reuptake inhibitors (SSRIs), has increased globally over the last few decades with the percentage of pregnant women users ranging between 1.2% and 6.2% up to 2005 [1,2,3,4]. SSRIs were considered to cause fewer side effects compared to the first generation of antidepressants until 2005, when a warning about the increased risk of foetal congenital heart anomalies (CHA) with SSRI use in pregnancy was released by the US Food and Drug Administration. This warning was shown to cause a decline, by 1.48 prescriptions per 1000 women per month, in the prescribing of SSRIs among pregnant women in the US and Canada between 2005 and 2007 [5]. Following this warning, many studies were carried out to evaluate the risk of congenital anomalies in children exposed to SSRIs during the first trimester of pregnancy. Most of these studies used data from healthcare monitoring systems and, while a large number of studies were done, the results have been inconsistent. Some studies reported an association, while other studies did not. With the emergence of genomic testing and personalized therapy, we now have the opportunity to explore the pharmacogenetic parameters that may explain why some children exposed to SSRIs develop a congenital heart anomaly and others do not. 

This review presents our current knowledge about the associations between serotonin reuptake inhibitors (SRIs) and CHA, about the pharmacogenetic predictors that are potentially involved in the pharmacokinetics of SRIs during pregnancy and about the genetic predictors involved in the plausible biological mechanisms linking CHA to SRIs exposure, taking into consideration maternal and foetal factors. We use the classification of serotonin reuptake inhibitors (SRIs) because it includes SSRIs and serotonin/noradrenaline reuptake inhibitors (i.e., venlafaxine and duloxetine), both of which are based on the same mechanism of serotonin inhibition.

## 2. The Risk of Congenital Heart Anomalies (CHA) Associated with Maternal Use of Serotonin Reuptake Inhibitors (SRIs) during the First Trimester of Pregnancy

To have an insight into the current knowledge about the association between maternal use of SRIs during pregnancy and the risk of CHA, we performed a literature search for cohort and case-control studies published between January 2005 and May 2015 using the PubMed database (Figure S1). Among 27 articles that were selected for review, no consistent pattern has been observed in the reported risk of CHA. A slight increase in risk was found, particularly for paroxetine, in a number of studies in various countries (see Table S1) [6,7,8,9,10,11,12,13,14,15,16,17,18,19,20,21,22], but a number of other studies reported no increased risk (see Table S2) [23,24,25,26,27,28,29,30,31,32]. The dose of SRIs may also be an important determinant of the risk. A dose–effect relationship was observed for paroxetine in one study [21], but it was not replicated in a subsequent study [33]. The results of a meta-analysis by Wurst and colleagues in 2010 indicate an increased prevalence of cardiac malformations (odds ratio (OR) 1.46, 95% confidence interval (CI) 1.17–1.82) after paroxetine use during the first trimester of pregnancy [34]. Another meta-analysis by Grigoriadis and colleagues in 2013, using adjusted data and excluding studies below a specified quality threshold, has also reported a significantly higher risk of cardiovascular malformations after maternal paroxetine use (risk ratio (RR) = 1.43, 95% CI 1.08–1.88) [35]. Similar findings were also reported in a meta-analysis performed by Myles and colleagues in 2013 (OR 1.44, 95% CI 1.12–1.86) [36]. The most recent meta-analysis, performed in 2015 including only prospective cohort studies, however, found no association of first trimester exposure to overall SRIs with an increased risk of CHA [37].

Most studies are population-based, linking drug exposure data from prescription databases with foetal outcome data from hospitals or birth defect registries. This approach has many limitations because these cohorts were not designed to investigate the foetal outcome following exposure to specific drugs [8,17,18,21,23,24,25,29,31]. Consequently, many confounding factors cannot be addressed, and biases in exposure and outcome definitions have always been major considerations [38]. While there are no perfect studies, each represents a different population and different risk factor assessments, and the study designs have improved over the years. A recent Bayesian analysis by the National Birth Defects Prevention Study (NBDPS), based on the results of previous population-based studies and new NBDPS data, has reported that paroxetine and fluoxetine use during pregnancy were associated with a higher risk of several subtypes of CHA [39]. Paroxetine was associated with atrial septal defects (ASDs) with posterior OR 1.8, 95% credible interval (CrI) 1.1–3.0 and right ventricular outflow tract obstruction defects (RVOTO) (posterior OR 2.4, 95% CrI 1.4–3.9). Fluoxetine was also associated with RVOTO (posterior OR 2.0, 95% CrI 1.4–3.1) and ventricular septal defects (VSDs) (posterior OR 1.4, 95% CrI 1.0–1.9). Although VSDs and ASDs are the most common subtypes of CHA (34% and 13%, respectively, of total CHA cases worldwide) [40], the absolute risk among children who were exposed to both SSRIs may still be considered low. 

There are concerns among the patients who were taking these medications when they became pregnant, but there is still no definite answer if SRIs increase the risk of CHA in offspring. Because congenital heart anomalies are not common diseases (8/1000 live-borns babies), and the number of cases exposed to SRIs is low, this inevitably leads to difficulties in obtaining a large enough sample to prove an association. Patients’ worry about the risk may lead to noncompliance of SRIs among pregnant women, which may potentially cause serious consequences for their therapeutic management. The best practice at present is to assess the individual risk factors before prescribing SRIs to pregnant women. Studies on the pharmacogenetics of SRIs can contribute to the understanding of the variability in risk estimates of SRI-induced CHA, and may assist in identifying mothers who are at a higher risk of having a child with CHA. 

## 3. Pharmacogenetic Predictors of SRI Pharmacokinetics 

**Figure 1:**
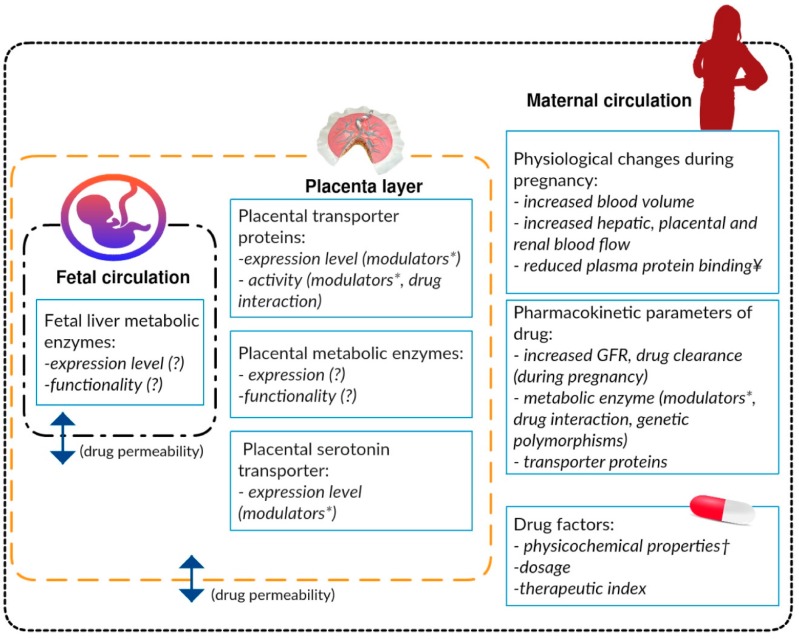
Factors influencing foetal drug exposure divided into factors in the maternal circulation, placenta layer and foetal circulation: *, substances that induce or inhibit the activity of enzymes/transporters, which may include pregnancy hormones and other drugs taken by the mother; ¥, important for serotonin reuptake inhibitors (SRIs) with high protein binding (e.g., fluoxetine, paroxetine, sertraline); † molecular size, polarity, charge, and lipophilicity of the drug; GFR, glomerular filtration rate; the double headed arrows indicate passive diffusion of drugs.

During pregnancy, the pharmacokinetics (absorption, distribution, metabolism and excretion) of SRIs are known to be altered because of the physiological changes associated with pregnancy. These changes include increased total body water (including blood volume), reduced albumin concentration (by up to 10 g/L and crucial for SRIs with high protein binding, e.g., fluoxetine, sertraline, paroxetine, duloxetine), modulation of metabolic enzymes by pregnancy hormones and increased renal function and drug clearance [41,42]. These physiological adaptations influence the level of SRIs in the maternal circulation, and subsequently affect the amount transferred to the foetus (Figure 1).

The passage and metabolism of SRIs supposedly occur through the yolk sac in the early stage of the first trimester up until the placenta forms in the late stage of the first trimester. Unlike other species, little is known about the transporters and binding proteins in the human yolk sac relevant for the availability and toxicity of chemicals to the embryo [43,44]. Nevertheless, drug transport in early pregnancy is postulated to be affected by pH gradients and protein binding between maternal and foetal compartments [44].

SRIs, with molecular weights around 300 g/mol, are able to cross the placenta, although the amount transferred in the first trimester is difficult to measure. In term placenta, the mean ratio of umbilical cord concentration to maternal serum concentration varies among SRIs depending on their molecular weight and polarity. The highest ratio was found for venlafaxine (range 0.72–1.1) and citalopram (0.71–0.83), followed by fluoxetine (0.64–0.73). The transfer of paroxetine and sertraline across the placenta seemed to be much lower (0.15–0.54 and 0.29–0.33, respectively) [45,46,47]. However, term data may not be representative of the first trimester of pregnancy.

### 3.1. Maternal Metabolic Enzymes

The most important enzymes in SRI metabolism are the cytochrome P450 (CYP) enzymes, including CYP1A2, CYP2B6, CYP2C9, CYP2C19, CYP2D6, and CYP3A4 isoenzymes. These enzymes are responsible for the inactivation of SRIs, and are mainly expressed in the maternal liver, with the exception of CYP3A4, which is also expressed in the small intestine [48]. In the placenta, mRNAs were found for CYP1A2, CYP2D6, CYP3A4, CYP3A5 and CYP3A7 in the first trimester, but their protein expression and functionality was not widely characterized. Meanwhile, for CYP1A1, the mRNA, protein and functional activity were detected during the first trimester, but not in subsequent trimesters [49,50]. In term placenta samples, high expression and functional activity were detected for CYP19A1, which is responsible for the conversion of androgens to oestrogens [51]. 

The metabolism of each SRI agent varies depending on its affinity towards the isoenzymes. Fluoxetine, paroxetine, venlafaxine and duloxetine are metabolized to a major extent by CYP2D6, and to a lesser extent by CYP1A2 (for duloxetine), CYP2C9 and CYP3A4 (for fluoxetine), and CYP2C19 and CYP3A4 (for venlafaxine) [52,53,54,55]. CYP2C19 is the major metabolic enzyme for citalopram and escitalopram (CYP3A4 and CYP2D6 to a lesser extent); CYP3A4 for sertraline (CYP2B6, CYP2C9, CYP2C19, CYP2D6 to a lesser extent); and CYP2D6, CYP1A2 and CYP3A4 for fluvoxamine [52,53,56]. Unlike other SRIs, fluoxetine is a prodrug that will be metabolized to an active enantiomer, norfluoxetine, to promote pharmacological action. As for CYP19A1, there is no data found for the metabolism of SRIs with this enzyme.

The knowledge of genetic variation of CYP enzymes has been used in practice for dose modification of certain drugs [57,58,59]. The polymorphisms of *CYP2C9*, *CYP2C19* and *CYP2D6* are well documented and cause changes in protein expression and function, leading to alterations in the plasma level of substrate drugs that consequently affect the clinical efficacy and toxicity (Table 1). A dosing guideline for SSRIs (paroxetine, fluvoxamine, citalopram, escitalopram and sertraline) for *CYP2D6* and *CYP2C19* genotypes was recently introduced [59] based on the results of numerous clinical and association studies [48,60]. Our great concern is for mothers with single nucleotide polymorphisms (SNP) leading to a poor metabolizer phenotype (i.e., CYP2D6*3/*4, *4/*4, *5/*5, *5/*6 or CYP2C19*2/*2, *2/*3, *3/*3), who are at a greater risk of SRI overdosing and side effects. The slower metabolism of SRIs leads to a greater concentration of these drugs in the mother’s bloodstream, which could lead to a higher concentration crossing the placental barrier. However, only a few studies have focused on the effect of CYP enzyme genotypes on the SRI pharmacokinetics during pregnancy. The maternal *CYP2D6* genotype of intermediate and poor metabolizers showed an increase in plasma concentration of paroxetine of 0.82 mg/L (95% CI 0.42–1.22) for each week over the course of pregnancy, which is in contrast to the decline observed among extensive and ultra-rapid metabolizers [61]. CYP2C9*2 and CYP2C9*3 were associated with a lower activity of CYP2C9 enzymes, which are thought to be responsible for the metabolism of fluoxetine, sertraline and venlafaxine, but these studies used minimal data and found a low strength of association [62,63]. Furthermore, the effect of genetic polymorphisms of *CYP1A2* has been studied less and is thought to contribute little to the pharmacokinetics of SRIs [60].

Apart from genetic polymorphisms, the inhibition or induction of CYP enzymes by certain drugs taken together with SRIs will also affect the metabolism of SRIs. For example co-medication with a CYP2D6 inhibitor was shown to be associated with increased plasma concentrations of citalopram, sertraline and venlafaxine, similar to the effect of the poor metabolizer phenotype [64]. 

### 3.2. Foetal Metabolic Enzymes

Little is known about the expression or activity of metabolic enzymes in the foetus. In the foetal liver, CYP3A7 has previously been reported as the dominant isoenzyme, and its expression decreases postnatally when it is substituted by CYP3A4 [65]. Genetic polymorphisms of *CYP3A4* contribute to a minor extent to drug pharmacokinetics and clinical therapy, including that of SRIs [66]. However, more recent evidence suggests high phenotypic inter-individual variability in foetal expression of CYP3A4 and CYP3A7, and that gestational age is not the most important covariate [67]. Foetal SNP CYP3A7*1E has been clinically demonstrated to reduce the efficacy of betamethasone in stimulating foetal lung maturity following maternal antenatal administration, although the exact mechanism remains unknown [68]. Meanwhile, in adult liver and intestinal cells, the interindividual variability in CYP3A7 expression was very pronounced, while the variant alleles of CYP3A7*1B and CYP3A7*1C were found to be associated with an increase in enzyme expression [65]. However, with regard to the metabolism of SRIs, there is no data so far indicating the role of CYP3A7 in the metabolism of these drugs. Although CYP2C9 and CYP2C19 were also shown to have functional activity in some foetal liver samples, there is a high variability in the expression profile between samples [69,70]. Among 60 foetuses aged less than 30 weeks of gestational age, CYP2D6 protein expression (5% as of adult) and functional activity (1% as of adult) was detected in only 30 of all liver samples [71]. Overall, the expression and activity of CYP2D6 in the first and second trimester foetal samples were either undetectable or very low, and the expression and activity increased in the third trimester [72]. In general, our knowledge of foetal metabolic enzymes is limited, and a high interindividual variability in the expression profile was observed. As the activity of these enzymes in the foetal liver may need further investigations, the contribution of these enzymes to the foetal metabolism of SRIs, particularly in the first trimester, is probably minor.

### 3.3. Placental Transporter Proteins 

The placenta expresses several transporter proteins that are involved in the regulation of the chemical environment of the foetus by transporting and removing toxic substrates [73,74,75]. Meanwhile, transporter proteins expressed in other organ cells, e.g., the intestine, kidney and liver, are important for the absorption, distribution and excretion of SRIs and their metabolites. One of the most-studied placental transporters is P-glycoprotein (P-gp), which is expressed in the maternal-facing membrane of the placental syncytiotrophoblast [76,77]. P-gp facilitates the efflux transport of a wide range of substrate drugs, including SRIs [78,79,80,81]. The expression of P-gp is highest in the early stages of pregnancy [82,83] denoting the role of P-gp in limiting the foetal exposure to xenobiotics or other harmful substances. Our previous study has shown that the inhibition of P-gp efflux activity of drug substrates was associated with an increased risk of congenital anomalies for drugs that were associated with certain types of congenital anomalies [84]. 

The polymorphisms of the *ABCB1* gene encoding for P-gp have been studied extensively with a focus on its effect on the pharmacokinetics, clinical efficacy and toxicity of antidepressants [85,86,87,88]. These studies focused on P-gp expression in the blood–brain barrier, which plays an important role in the bioavailability of these antidepressants in the brain. Under normal conditions, P-gp effluxes the substrates out of the brain cells, which can either lead to lower efficacy or reduced side effects of the substrates. Several *ABCB1* SNPs (*3435C>T*, *1236C>T*, *2677G>T*) previously associated with reduced P-gp expression, have also been associated with increased efficacy or increased side effects that lead to switching and discontinuation of therapy [89,90,91]. In the placenta, *3435C>T*, *1236C>T* and *2677G>T* SNPs were associated with a reduced mRNA and/or protein expression of P-gp in human placental samples, suggesting a weaker foetal protection against potential teratogens [92,93,94]. This finding was supported by two clinical studies that found an increased risk of cleft lip [95] and CHA [96] associated with a maternal *3435T* variant allele in mothers taking any medication during the first trimester of pregnancy. The risk was even higher in mothers who did not take folic acid supplements [95,96]. Several other *ABCB1* SNPs relevant to the pharmacogenomics of SRIs were found to be associated with SRI response and adverse events. In Table 1, the predicted effect on protein expression/activity in the placenta and the predicted effect on foetal exposure to SRIs are shown. 

Maternal metabolic CYP enzymes and placental transporters both play an important role in determining the foetal SRI exposure. Metabolic enzymes affect the concentration of SRIs in the maternal circulation, while placental P-gp determines the amount transported into the foetal circulation. Any changes in the expression and function of these enzymes and transporters may lead to variation in foetal SRI exposure. Despite the need to evaluate the extent of foetal SRI exposure, there are a limited number of ways to measure it directly, e.g., using animal studies and in vivo, in vitro or ex vivo placental transfer models [74,75,97]. When examining the genetic factors, one should take both the mother and the foetus into consideration as both provide several mechanisms to limit foetal exposure to SRIs.

## 4. Pharmacogenetic Predictors of CHA Associated with Exposure to SRIs

Serotonin (5-HT) is a neurotransmitter that also acts as a growth factor and is an important regulatory factor during a critical period of embryo development. The period of about 20–70 days following fertilization involves the formation of the brain [43,116]. The foetal heart also undergoes gross morphological changes within the first 112 days of development, including septation (between 35 and 53 days), formation of the valve components (between 49 and 56 days) and delamination of the leaflets into the tricuspid valve (between 56 and 112 days) [117]. The cardiac morphogenesis is dependent on the migration, survival and proliferation of neural crest cells, which are regulated by 5-HT, mainly via the 5-HT2B receptor [118,119]. 5-HT is also one of the factors in the signalling cascade driving the establishment of laterality in heart cells. Disruptions in the laterality cascade result in laterality defects of the heart such as atrial isomerism, transposition of the great arteries, double outlet right ventricle and common truncus arteriosus [120]. The pathology of heart defects has also been postulated to be associated with the pattern of intracardiac blood flow [121], which is another link between 5-HT and heart development because 5-HT acts as a potent vasoconstrictor and is important in maintaining an optimal uteroplacental blood flow [122].

During embryogenesis, the embryo is supplied with 5-HT from the maternal blood. 5-HT in the maternal circulation can be transported to the foetal circulation by the serotonin transporter (SERT) expressed in the placenta, and signals through serotonin receptors in the foetus [123]. However, in depressed mothers, there is an abnormally reduced function of the serotonergic system in the brain. It is commonly agreed that for women who took antidepressants during pregnancy, the effect on foetal outcome is difficult to measure and disentangle from the effect of depression itself, since there is a lack of evidence to conclude whether depression itself poses an increased risk for CHA [8,124,125]. Therefore, we are looking for other possible factors, for instance the polymorphisms of SERT and foetal serotonin receptors that might possibly be among the predictors of the risk of CHA.

### 4.1. Serotonin Transporter in Foetal Cardiac Cells and in the Placenta 

Based on animal and in vitro studies, the effect of SRIs on embryonic heart development can occur via modulation of serotonin transporter levels and prenatal 5-HT levels [126,127]. In humans, this effect occurs via direct exposure to SRIs, which are readily passed through the placenta, to the foetal serotonergic system. In the foetus, SRIs inhibit SERT expressed in the foetal cardiac cells, which subsequently reduce the transport of 5-HT into the cells and could, in theory, disturb the normal development of the heart. In addition, SRIs can also inhibit SERT expressed in the placenta, which will limit the transport of 5-HT and/or other important growth factors through the placenta for foetal use [116]. 

Polymorphisms of the *SLC6A4* gene encoding for SERT may also play a role in the serotonin signalling in foetal heart development. The genetic variation in the SERT promoter gene region, SERTPR (formerly 5-HTTLPR), was previously associated with SRI response and adverse risk events (Table 2). This insertion/deletion polymorphism includes a short (S) and a long (L) allele, and the S allele is associated with reduced activity in placental tissue and increased risk of adverse neonatal outcome events associated with SRI use [128,129]. Another polymorphism, rs25531 is putatively located in the sixth repeat of the SERTPR, with L_A_ or L_G_ alleles. The expression of SERT is known to be higher in the L_A_ allele, while it is reduced in the L_G_ allele to a level similar to the SERTPR S allele [130]. Since the SRIs inhibit SERT, less expression of this transporter may increase the inhibition rate. That is, foetuses with S or L_G_ genotype are likely to receive a higher “effective” dose, considering there is less SERT to be blocked. As a consequence, a lower amount of 5-HT is permitted into the foetal circulation [128] to regulate normal cardiac morphogenesis.

### 4.2. Foetal Serotonin Receptors 

5-HT activates seven distinct families of 5-HT receptors with 16 subtypes, and most of the receptors are G-protein coupled [131]. Several SNPs of genes encoding for 5-HT1A, 1B, 2A and 3B were reported to be correlated with SRI response and side effects, which might be related to the alteration in receptor expression or activity in the nervous system. Some polymorphisms were associated with a better response to SRIs, for example, of the *HTR2A rs6314*, *rs1928040*, *rs7997012*, *rs6311* [132,133,134,135,136,137], and of the *HTR1A rs1364043* and of the *HTR1B rs6296* in the treatment of citalopram [138]. Other polymorphisms, on the other hand, were shown to reduce the response of several SRIs, e.g., *HTR1A rs6295* in the treatment of fluoxetine, fluvoxamine, and citalopram [138,139,140,141] Furthermore, an increase in side effects of paroxetine was reported among patients with *HTR2A rs6313*, *HTR3B rs1176744* and *HTR3B rs3831455* [132,142,143]. Unlike other genes, there are limited data on the polymorphisms of the gene encoding for the 5-HT2B receptor, which is more important, in this regard, in the developmental stage of the foetal heart [118,119,144].

When a woman in the first trimester of pregnancy is required to take SRIs, we can assume a reduced amount of 5-HT may be transferred into the foetal circulation following the inhibition of placental SERT. The reduced concentration of 5-HT in the foetal circulation, together with the changes in the expression and/or activity of the 5-HT receptors, may subsequently affect the normal development of the foetal heart. 

### 4.3. Other Genes 

Most CHA have a complex aetiology, with some caused by a Mendelian trait or a chromosomal aberration. The genetic aetiology of CHA is not yet well understood, and the known genetic causes of CHA account for less than 20% of CHA cases [121,145] The genetic variations of other genes involved in the pathway of foetal heart development are not emphasized in this review, but should also be taken into consideration in determining the true causal relationship between SRI exposure and CHA. A recent study found that the placenta of SSRI-treated mothers had a lower expression of the *ROCK2* gene, which is thought to play a role in the development of the cardiovascular system of the foetus, as compared to untreated depressed and healthy mothers [125]. In contrast, a study with a similar setting, but focused on the neurotrophic growth factor signalling pathway, found an increased level of the *ROCK2* gene and of phosphorylated ROCK2 in SSRI-treated women in comparison to depressed and healthy women [146]. Despite the contrary findings, both studies speculated that ROCK2 expression could be altered in the placenta of SSRI-treated women, and might disturb the normal development of the foetal cardiovascular system. Another aspect to be considered is the effect of foetal epigenetic programming, which is currently being investigated as the candidate molecular mechanism underlying physiological alterations in exposed foetuses [125].

Our understanding of the biological plausibility, corroborated by the evidence, may indicate that prenatal use of SRIs causes an alteration in the signalling pathway important for the development of the foetal heart. This alteration is also dependent on the pharmacokinetics of SRIs in maternal and foetal circulation. Moreover, any alteration in the expression and/or function of the enzymes, proteins, transporters and receptors involved in the signalling, modulated by the genetic polymorphisms, may theoretically alter the risk of CHA.

## 5. Conclusions

The scope of research on the risk of CHA associated with prenatal exposure to SRIs should be extended to include the role of pharmacogenetics in pregnancy. While implementing the results in clinical practice may still seem a distant prospect, we need to begin developing theories and doing model simulations that will help us understand the complex interactions between maternal and foetal genetics and their effect on foetal SRI exposure and the risk of CHA. A better understanding of these interactions is a crucial step toward considering personalized drug treatment models for pregnant women with depression.

## Figures and Tables

**Table 1:** Overview of polymorphisms significantly associated with serotonin reuptake inhibitors (SRIs) pharmacokinetics and their predicted effect on foetal SRI exposure.

Gene	SNPs	rs Numbers	MAF (%) ^a^			Pharmacokinetics and/or Clinical Effects	Phenotype (Predicted Expression/Activity of CYP Enzymes/Transporter Proteins)	Predicted Effect on Foetal SRI Exposure ^b^	SRIs Likely to Be Affected
			Caucasians	Asians	Africans				
*CYP1A2*	*−3113G>A*	rs2069521	3	8	11	Increased severity of side effects of escitalopram [98]	Increased ^c^	Reduced	Fluvoxamine, duloxetine
	*−10 + 103 T>G*	rs2069526	3	8	12	Increased severity of side effects of escitalopram [98]	Increased ^c^		
	*832 − 249 C>T*	rs4646425	3	8	0	Increased severity of side effects of escitalopram [98], reduced efficacy of paroxetine [99]	Increased^c^		
	*1253 + 81 T>C*	rs4646427	3	8	11	Increased severity of side effects of escitalopram [98]	Increased ^c^		
	*1042 + 43 G>A*	rs2472304	59	16	4	Increased efficacy of paroxetine [99]	Reduced ^c^	Increased	
	*1548C>T*	rs2470890	59	16	3	Increased efficacy of paroxetine [99]	Reduced^c^		
*CYP2C9*	*2	rs1799853	11	0	4	Reduced metabolism of fluoxetine [62,63]	Reduced ^d^	Increased	Fluoxetine, sertraline, venlafaxine
	*3	rs1057910	7	3	2	Reduced metabolism of fluoxetine [62,63]	Reduced ^d^	Increased	
*CYP2C19*	*2	rs4244285	15	33	17	Reduced tolerance to citalopram [100] and reduced metabolism of escitalopram [101]	Reduced ^c,d^	Increased	Citalopram *, escitalopram *, sertraline, venlafaxine
	*3	rs4986893	0	5	0	Reduced metabolism of escitalopram [102]	Reduced ^d^	Increased	
	*17	rs12248560	23	2	22	Increased metabolism of citalopram [103], escitalopram [102,104]	Increased ^d^	Reduced	
*CYP2D6*	*3	rs35742686	2	0	0	Reduced metabolism of escitalopram [104], venlafaxine [105]	No activity ^d^	Increased	Paroxetine *, fluoxetine *, venlafaxine *, fluvoxamine, sertraline
	*4	rs3892097	19	0	6	Reduced metabolism of escitalopram [104], venlafaxine [105,106]	No activity ^d^	Increased	
	*5	whole gene deletion	4	7.2	ND	Reduced metabolism of paroxetine [107]	No activity ^d^	Increased	
	*10	rs1065852	20	52	9	Reduced metabolism of paroxetine [107]	Reduced ^d^	Increased	
*ABCB1* (P-gp)	*3435C>T*	rs1045642	53	40	15	Increased efficacy of escitalopram [108,109], venlafaxine [109], increased concentration of fluvoxamine [110], a group of antidepressants [89]	Reduced ^c,d^	Increased	Paroxetine, fluoxetine, venlafaxine, fluvoxamine, sertraline, venlafaxine, citalopram, escitalopram
	*1236C>T*	rs1128503	43	66	14	Increased concentration and side effects of antidepressants [89]	Reduced ^c,d^	Increased	
	*3489 + 1573G>A*	rs1882478	26	57	63	Increased efficacy of escitalopram [108]	Reduced ^c^	Increased	
	*2677G>T*	rs2032582	43	45	3	Reduced concentration and efficacy of citalopram [111], increased efficacy of paroxetine [90]	Increase or reduced ^c,d^	Increased or reduced	
	*2493 + 49T>C*	rs2035283	13	6	22	Increased efficacy of paroxetine [112] and side effects of SSRIs [113]	Reduced ^c^	Increased	
	*2481 + 24G>A*	rs2235040	13	6	20	Increased efficacy of paroxetine [112] and side effects of SSRIs [113]	Reduced ^c^	Increased	
	*2482 − 236A>G*	rs4148739	13	6	22	Increased efficacy of SSRIs [114]	Reduced ^c^	Increased	
	*61A>G*	rs9282564	9	0	0	Increased efficacy of paroxetine [115]	Reduced ^c^	Increased	
	*287 − 1234G>C*	rs10256836	29	15	8	Reduced efficacy of escitalopram [108]	Increased ^c^	Reduced	
	*2927 + 314G>A*	rs28401781	13	6	20	Increased efficacy of SSRIs [114]	Reduced ^c^	Increased	

Abbreviations: MAF, minor allele frequency; ND, no data; CYP, cytochrome P450; P-gp, P-glycoprotein; SSRIs, selective serotonin reuptake inhibitors. * Causes dose modification in patients with polymorphic variants [57,58,59]; ^a^ MAFs from SNPedia, www.cypalleles.ki.se, PharmGkb, 1000 Genomes, HapMap; ^b^ predicted effect on foetal SRI exposure: the exposure is predicted to be increased if the expression/activity of CYP enzymes is reduced, leading to an increase in SRI concentration in the maternal circulation and more SRI transported through the placenta (and vice versa); ^c^ based on clinical data; ^d^ based on pharmacokinetic data.

**Table 2:** Polymorphisms of the serotonin transporter (SERT) and their predicted effect on congenital heart anomalies (CHA) risk in offspring exposed in utero.

Gene	SNPs	rs Numbers	MAF (%) ^a^			Clinical Effects	Phenotype (Predicted Enzyme/Protein Expression or Activity)	Predicted Effect on CHA Risk ^b^	SRIs Likely to Be Affected
			Caucasians	Asians	Africans				
*SLC6A4* (SERT)	*SERTPR* or *5-HTTLPR* (S and L alleles)	rs4795541	40 (S)	80 (S)	17 (S)	S-allele: poor response to venlafaxine [147], fluoxetine [139,148], increase side effects of fluvoxamine [137], citalopram [149], escitalopram [150], paroxetine [151] and overall SSRIs [152,153]	Reduced with S allele	Increased	Fluoxetine, citalopram, sertraline, paroxetine, escitalopram, fluvoxamine
	*−1936A>G* (*SERTPR* L_A_/L_G_ allele)	rs25531	9	8	21	L_G_ allele: increased risk of side effects and poor response citalopram [149] and overall SSRIs [153]	Reduced with L_G_ allele	Increased	
	*5HTT VNTR* (9,10 or 12 repeat)	rs57098334	47 (10)	10 (10)	26 (10)	12 allele was associated with higher rates of side effects of SSRIs [153]	Increased transcription with 12 repeats	Reduced	

Abbreviations: MAF, minor allele frequency; S, short allele; L, long allele. ^a^ MAFs from SNPedia, www.cypalleles.ki.se, PharmGkb, 1000 Genomes, HapMap; ^b^ predicted effect on CHA risk: based on hypothetical conditions (see text).

## Supplementary Materials

Supplementary materials can be found at www.mdpi.com/1422-0067/17/8/1333/s1.
