# Supplementary material for: The Risk of Congenital Heart Anomalies Following Prenatal Exposure to Serotonin Reuptake Inhibitors—Is Pharmacogenetics the Key?
Source: Int J Mol Sci. 2016 Aug 13;17(8):1333. doi: 10.3390/ijms17081333 (PMC5000730; doi:10.3390/ijms17081333)
Supplement: Supplementary file 1 [file ijms-17-01333-s001.pdf]

Supplementary Materials: The Risk of Congenital Heart Anomalies Following Prenatal Exposure to Serotonin Reuptake Inhibitors—Is Pharmacogenetics the Key?

Aizati N. A. Daud, Jorieke E. H. Bergman, Wilhelmina S. Kerstjens-Frederikse, Henk Groen and Bob Wilffert

Supplementary Materials

This Word document summarizes the studies published from 2005 through 2015 that either do (Table S1) or do not (Table S2) find an association between the use of serotonin reuptake inhibitors (SRIs) during early pregnancy and the risk of congenital heart anomalies (CHA) and/or other congenital anomalies (CA).

Search Strategy

A literature review was performed in May 2015 using the PubMed database. Articles are searched using the combinations of the following keywords: “Serotonin Uptake Inhibitors” [Mesh], “Congenital Abnormalities” [Mesh], and “humans” [MeSH Terms]. See Supplementary Figure S1 for the search strategy outline.

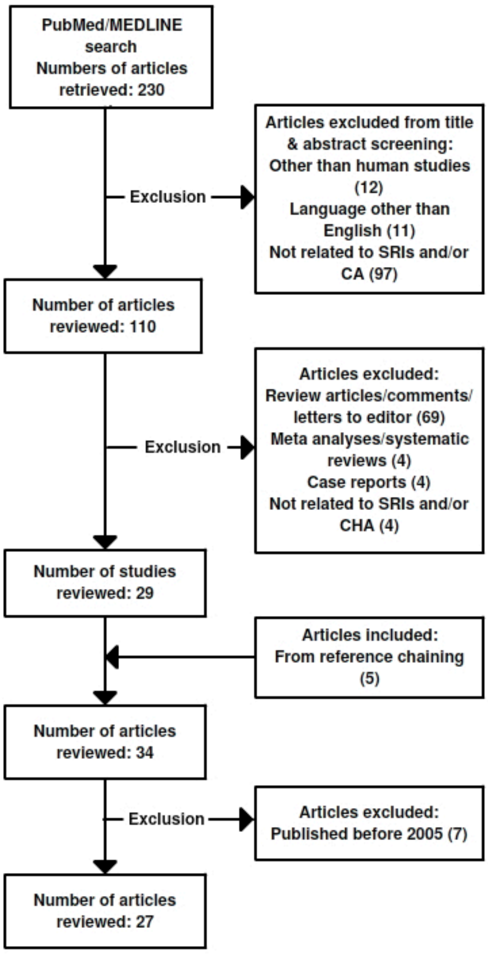

Figure S1. Flow-chart outlining the search strategy. SRIs, serotonin reuptake inhibitors; CA, congenital anomalies; CHA, congenital heart anomalies.

Table S1. Studies (2005–2015) that found an association between SRI use in early pregnancy and the risk of congenital anomalies including CHA.

Authors	Study Design (Database Used)	Location	Number of Patients	SRIs/SSRIs	Definition of First Trimester Exposure	Outcome (Anomalies) Definition	Drawn Conclusion
Be´rard et al., 2015 [6]	Cohort study (pregnancy cohort)	Canada	18,493 pregnancies (2329 exposed to SRIs)	Sertraline & other SSRIs	14 weeks of the first trimester (confirmed with ultrasound)	Major congenital anomalies detected within the first year of life	Infants exposed to sertraline were at increased risk of ASD and VSD
Wemakor et al., 2015 [7]	Case-malformed control study (congenital anomaly registries)	12 European countries	42,983 cases and malformed controls (including TOP, miscarriages, stillbirths), 12,876 CHA cases	Any SSRIs without any other type of antidepressants	First day of the LMP up to 12th week of gestation	Major CHA (excluding preterm deliveries with only PDA and all cases with open foramen ovale)	Result supports teratogenic effects of SSRIs specific to certain anomalies, but cannot exclude confounding by indication or associated factors
Ban et al., 2014 [8]	Cohort study (primary care records)	UK	325,294 women without depression, 7683 women exposed to SSRIs	SSRIs alone, SSRIs + TCAs	1 month before to 3 months after conception	Major congenital anomalies, overall and specific	Paroxetine increases the risk of CHA. The risk of overall major congenital anomalies did not increase with maternal depression & antidepressants
Knudsen et al., 2014 [9]	Cohort study (birth defects registry)	Denmark	845 exposed, 71,435 not exposed	SSRIs	30 days before LMP until 91 days after LMP	CHA detected within first 5 years of life (excluding chromosomal anomalies, genetic syndrome or microdeletion)	SSRI use increases the risk of severe CHA and socioeconomic status did not confound the risk
Polen et al., 2013 [10]	Case-control study (NBDPS)	USA	27,045 women. Exposed: 14/8002 (0.17%) of controls, 77/19,043 (0.4%) of cases	Venlafaxine	1 month before conception & first trimester (maternal interview after delivery)	30 selected birth defects	Associations found for certain birth defects, especially anencephaly, cleft palate, gastroschisis, and some CHD (ASD, coarctation of aorta, LVOTO)
Malm et al., 2011 [11]	Retrospective cohort (national birth registers)	Finland	6881 exposed, 618,727 not exposed	SSRIs	1 month before pregnancy & first trimester (based on LMP and U/S data)	Major congenital anomalies (does not exclude chromosomal abnormalities)	Associations found for specific CV anomalies; fluoxetine & isolated VSD, paroxetine & right ventricular outflow defect

Table S1. *Cont.*

Authors	Study Design (Database Used)	Location	Number of Patients	SRIs/SSRIs	Definition of First Trimester Exposure	Outcome (Anomalies) Definition	Drawn Conclusion
Reis and Kallèn, 2010 [12]	Case-control study (national birth registers)	Sweden	14,821 exposed, 1,062,190 not exposed	Antidepressants	Early use: ~10–12 weeks, Later use: subsequent prescription	'Relatively severe malformations' excluding chromosomal abnormalities	Associations found between paroxetine and cardiovascular defects, and between SSRI and hypospadias (particularly with paroxetine)
Bakker et al., 2010 [13]	Case-malformed control study (birth defect registry)	Netherlands	678 cases of isolated heart defects, 615 controls of chromosomal anomalies	Paroxetine	4 weeks before conception until 12th week of pregnancy	Major isolated CHA	Increased risk of isolated ASD with paroxetine use in the first trimester, but the absolute risk is small
Merlob et al., 2009 [14]	Prospective study (birth defect surveillance database)	Israel	67,871 infants (235 exposed, 2537 not exposed)	SSRIs	First trimester (based on maternal interview upon admission to maternity ward)	Cardiac murmur on the first day of life and persist on 2nd and 3rd day	Increased risk of mild, non-syndromic heart defects in infants exposed to SSRIs (small risk)
Pedersen et al., 2009 [15]	Cohort study (national health & birth defect registries)	Denmark	1370 exposed, 493,113 not exposed	SSRIs (two or more filled Rx)	28 days before to 112 days after gestation.	CHA detected within the first year of life (exclude stillbirth and multiple births)	Citalopram and sertraline were associated with an increased prevalence of septal heart defects (limited risk)
Diav-Citrin et al., 2008 [16]	Prospective study (multi-centre TIS)	Israel, Italy, Germany	463 exposed to paroxetine, 346 exposed to fluoxetine, 1467 control: exposed to non-teratogens	Paroxetine and fluoxetine	Paroxetine: between weeks 3–13, fluoxetine: between weeks 2–13 after LMP	Major congenital anomalies, including VSD, detected within the first 6 years of life	Possible association between CV anomalies and first trimester exposure to fluoxetine
Oberlander et al., 2008 [17]	Cohort study (national health registries)	Canada	2625 exposed, 107,320 not exposed	SRIs	Days of dosing covered by the Rx overlapped with the period from LMP to LMP + 90 days	Major congenital anomalies	The risk for cardiac anomalies increased when SRIs were used in combination with benzodiazepine (possible effect of drug interaction)

Table S1. *Cont.*

Authors	Study Design (Database Used)	Location	Number of Patients	SRIs/SSRIs	Definition of First Trimester Exposure	Outcome (Anomalies) Definition	Drawn Conclusion
Cole et al., 2007 [18]	Cohort study (insurance claim database)	USA	paroxetine: 791 (monotherapy), 989 (mono or polytherapy); other antidepressants: mono (4072), mono & poly (4767)	Paroxetine mono- or poly therapy	Prescription duration overlapping with the earliest conception date until 91 days	All major congenital malformations detected within the first 9 months of age	Increased risk of overall congenital anomalies with paroxetine exposure during the first trimester compared to the use of other antidepressants
Louik et al., 2007 [19]	Case-control study (birth defects surveillance database)	USA	9849 infants with and 5860 infants without birth defects	SSRIs	28 days before to 112 days after the LMP	Overall and specific anomalies, major only	Increased risk for some specific anomalies with individual SSRIs use (small absolute risks)
Källén et al., 2007 [20]	Cohort study (national birth registries)	Sweden	6481 exposed, 873,876 not exposed	SSRIs	Detected during the first antenatal visit (90% before the end of week 12)	Overall anomalies (including minor conditions)	Paroxetine increased the risk of any cardiac anomalies, mostly of ASD & VSD
Berard et al., 2007 [21]	Case-control study (national health registries)	Canada	1403 women. 542 exposed to paroxetine,443 to other SSRIs, 418 to other antidepressants	Paroxetine	The first trimester of pregnancy (0–14 weeks of gestational age)	Any major anomaly including cardiac anomalies	Association found only in doses above 25 mg/day
Wogelius et al., 2006 [22]	Cohort study (national birth registry)	Denmark	1051 exposed, 150,780 not exposed	SSRIs	30 days before conception until the end of the first trimester	Congenital anomalies detected within the first year of life	A moderately increased risk of overall congenital anomalies was found with the use of SSRIs

Abbreviations: SRI, serotonin reuptake inhibitor; SSRI, selective serotonin reuptake inhibitor; CHA, congenital heart anomalies; ASD, atrial septal defect; VSD, ventricular septal defect; TOP, termination of pregnancy; LMP, last menstrual period; PDA, patent ductus arteriosus; NBDPS, the National Birth Defects Prevention Study; LVOTO, left ventricular outflow tract obstruction; U/S, ultrasound; TIS, Teratology Information Service.

Table S2. Studies (2005–2015) reporting no association between SRI use in early pregnancy and the risk of congenital anomalies including CHA.

Authors	Study Design	Location	Number of Patients	SSRIs	Definition of First Trimester Exposure	Outcome (Anomalies) Definition	Drawn Conclusion
Furu et al., 2015 [23]	Cohort study and sibling design (national health registries)	Denmark, Finland, Iceland, Norway, and Sweden	2.3 million live singletons, 2288 sibling cohort	All SSRIs, including venlafaxine	30 days before the first day of LMP until the end of the first trimester	Major cardiac and other anomalies diagnosed within 1 year after birth	Results did not suggest teratogenic effect of SSRIs and venlafaxine
Huybrechts et al., 2014 [24]	Cohort study (national health registry)	USA	46,144 exposed to SSRIs, 885,115 not exposed	SSRIs and other antidepressants	Days/duration of Rx supplied overlap with 90 days the first trimester	Any cardiac malformations	Do not support earlier findings of an association between antidepressants & cardiac anomalies
Vasilakis-Scaramozza et al., 2013 [25]	Matched cohort study (general practice records)	UK	3276 exposed, 6617 non-exposed (singleton pregnancies)	TCA and SSRIs	At least one Rx of TCA or SSRIs from 180 to 335 days prior to delivery date for livebirth cases, and 70–225 days for stillbirths/TOP	Major congenital anomalies, detected before 1st birthday	Exposure to TCA or SSRIs does not increase the risk of congenital anomalies
Margulis et al. 2013 [26]	Cohort study (national livebirth cohort)	UK	3046 exposed, 8991 not exposed	SSRIs	First trimester (assumption of pregnancy duration 273 days, for term, 258 days for preterm births)	Cardiac malformation detected in the first year 6 years of life	No association found between maternal use of SSRIs and cardiac anomalies
Klieger-Grossmann et al., 2012 [27]	Observational cohort study (pregnancy surveillance registries)	Canada	6582 mothers (212 mothers in each group of escitalopram users, other antidepressants, and non-teratogenic drug users)	Escitalopram	The first trimester of pregnancy	Major congenital malformations	Escitalopram was not associated with increased risk for major malformation
Einarson et al., 2009 [28]	Matched-cohort study (teratogen information service)	Canada	928 each in the exposed and unexposed groups	Antidepressants (SSRIs, SNRIs)	First trimester (based on maternal interview)	Major congenital malformations (maternal interview, corroborated by physician report)	No increase in the risk of major malformations with the use of antidepressants as a group or individually

Table S2. *Cont.*

Authors	Study Design	Location	Number of Patients	SSRIs	Definition of First Trimester Exposure	Outcome (Anomalies) Definition	Drawn Conclusion
Wichman et al., 2009 [29]	Retrospective cohort study (health registry)	USA	808 exposed, 24,406 not exposed	SSRIs, venlafaxine	0–13 weeks of gestation	CHA (diagnosed at birth or before discharge), obstetric data and medical record	No associations between SSRI use and CHA
Lennestal et al., 2007 [30]	Cohort study (national birth registries)	Sweden	860,215 deliveries (732 women used SNRI/NRI in early pregnancy)	SNRIs/NRIs (mianserin, mirtazapine, venlafaxine, reboxetine)	First trimester (interview during the first antenatal care visit)	Delivery outcome, including congenital anomalies	No increase in the risk of congenital anomalies in infants exposed to SNRI/NRIs
Davis et al., 2007 [31]	Retrospective cohort study (health registry)	USA	1047 exposed to SSRI at any time during pregnancy, 75,833 not exposed to any antidepressants	SSRI, TCAs	First 90 days of pregnancy (assumption of gestational age of 270 days before delivery date)	Congenital malformations & perinatal complications	No increase in the risk of cardiovascular anomalies with paroxetine use
Alwan et al., 2007 [32]	Case-control study (NBDPS)	USA	9622 cases of congenital anomalies, 4092 controls. 408 exposed to SSRIs from both group	Any SSRIs	1 month before to 3 months after conception (date of conception: 266 days before the estimated date of delivery)	Major CHA, isolated and multiple anomalies	Maternal use of SSRIs was not associated with an increased risk of CHA or other birth defects

SRI: serotonin reuptake inhibitor; SSRI: selective serotonin reuptake inhibitor; CHA: congenital heart anomalies; LMP: last menstrual period; VSD: ventricular septal defect; TCA: tricyclic antidepressants; TOP: termination of pregnancy; SNRI: serotonin/noradrenaline reuptake inhibitor; NRI: noradrenaline reuptake inhibitor.
